# Effects of different botanical oil meal mixed with cow manure organic fertilizers on soil microbial community and function and tobacco yield and quality

**DOI:** 10.3389/fmicb.2023.1191059

**Published:** 2023-05-25

**Authors:** Yuxuan Chen, Xiaolin Lv, Yanmin Qin, Deping Zhang, Chaoqun Zhang, Zhanfeng Song, Dongyang Liu, Lianqiang Jiang, Bin Huang, Jie Wang

**Affiliations:** ^1^Pest Integrated Management Key Laboratory of China Tobacco, Tobacco Research Institute of Chinese Academy of Agricultural Sciences, Qingdao, China; ^2^Jiangxi Provincial Tobacco Company, Fuzhou, China; ^3^Guangxi Provincial Tobacco Company, Nanning, China; ^4^Sichuan Provincial Tobacco Company Liangshanzhou Company, Liangshanzhou, China

**Keywords:** cow manure, botanical oil meal, soil microorganism, community structure, function prediction

## Abstract

**Introduction:**

The continuous application of cow manure in soil for many years leads to the accumulation of heavy metals, pathogenic microorganisms, and antibiotic resistance genes. Therefore, in recent years, cow manure has often been mixed with botanical oil meal as organic fertilizer applied to farmland to improve soil and crop quality. However, the effects of various botanical oil meal and cow manure mixed organic fertilizers on soil microbial composition, community structure, and function, tobacco yield, and quality remain unclear.

**Methods:**

Therefore, we prepared organic manure via solid fermentation by mixing cow manure with different oil meals (soybean meal, rape meal, peanut bran, sesame meal). Then, we studied its effects on soil microbial community structure and function, physicochemical properties, enzyme activities, tobacco yield and quality; then we analyzed the correlations between these factors.

**Results and discussion:**

Compared with cow manure alone, the four kinds of mixed botanical oil meal and cow manure improved the yield and quality of flue-cured tobacco to different degrees. Peanut bran, which significantly improved the soil available phosphorus, available potassium, and NO_3_^–^-N, was the best addition. Compared with cow manure alone, soil fungal diversity was significantly decreased when rape meal or peanut bran was combined with cow manure, while soil bacterial and fungal abundance was significantly increased when rape meal was added compared with soybean meal or peanut bran. The addition of different botanical oil meals significantly enriched the *subgroup_7* and *Spingomonas* bacteria and *Chaetomium* and *Penicillium* fungi in the soil. The relative abundances of functional genes of xenobiotics biodegradation and metabolism, soil endophytic fungi, and wood saprotroph functional groups increased. In addition, alkaline phosphatase had the greatest effect on soil microorganisms, while NO_3_^–^-N had the least effect on soil microorganisms. In conclusion, the mixed application of cow manure and botanical oil meal increased the available phosphorus and potassium contents in soil; enriched beneficial microorganisms; promoted the metabolic function of soil microorganisms; increased the yield and quality of tobacco; and improved the soil microecology.

## 1. Introduction

Cow manure, as an organic fertilizer, has been widely used in agriculture because of its high utilization rate, good fertilizer efficiency, and low cost (Yang and Zhang, 2022). Economic globalization has promoted the expansion of intensive livestock breeding, producing increasing volumes of rich manure. If not handled properly, the odor, harmful gases, and dust-carrying pathogenic microorganisms produced by cow manure decomposition would endanger human health and the environment (Ginebra et al., 2022). In addition, applying cow manure for many years easily causes heavy metal accumulation in soil. If not treated properly before application, the remaining harmful bacteria would disrupt the ecological balance of soil microorganisms and threaten soil security (Hilaire et al., 2022). Furthermore, the residual antibiotics (such as tetracycline and sulfanilamide antibiotics) in cow manure can remain in soil for several years, leading to the evolution of antibiotic resistance genes in pathogenic bacteria, thereby increasing the abundance of antibiotic resistance genes in soil and damaging soil health (Du et al., 2022; Fuchs et al., 2022).

Oil meal is a by-product of oil extraction from crops—including soybean, rapeseed, peanut, and sesame—which produced from various plants, has high nutritional value. Compared with common fertilizer, the return of oil meal to the field through fermentation can effectively promote the release of nutrients in soil and reduce nutrient loss (Siva et al., 2021). Oil meal is rich in organic matter that can effectively improve soil fertility, and has rich nitrogen, phosphorus, and potassium contents that can strengthen roots; promote the development of crop roots; improve crop immunity and resistance; and effectively inhibit the infection of root bacteria. It has been reported that neem seed oil meal can inhibit the growth of *Candida albicans* when the concentration is 100–150 mg/L (Aramwit et al., 2022). Mahalik et al. (2020) found that 1 ton/ha of neem seed oil meal could effectively reduce the number of nodules (82.6%), egg masses (84.8%), and nematodes (66.3%) in soil, and increase the length and weight of cucumber roots and branches.

Several studies have shown that the mixed application of manure with plant resources from different sources can improve plant yield, quality, and disease resistance and enhance soil fertility (Ibrahim et al., 2018; Li et al., 2021). Firstka et al. (2017) found that the combined application of chicken manure and palm waste could provide higher amounts of plant essential nutrients (eg., P, K, Ca, Mg) and increase soybean yield in tropical-aged soils. Tian and Zheng found that compost tea (a mixture of pine bark, manure, and earthworm dung) had inhibitory effects on *Fusarium*, *Rhizoctonia reguckus*, *Rhizoctonia oryzae*, and *Phytophthora* in vitro (Tian and Zheng, 2013). Cow manure is mixed with straw to increase its aeration, self-heating, and insulation properties to enhance the death of pathogens and thus reduce the risk of environmental transmission when manure is applied to land (Patricia et al., 2014). Botanical oil meal is also used in combination with other agricultural waste to enhance its fertility. It has been reported that the combination of oil meal with fertilizer, beneficial microorganisms, and other auxiliary materials can improve soil microbial activity, promote nutrient cycling, and effectively solve the problem of continuous cropping obstacles (Liu et al., 2018). Huang et al. (2021) found that mixed fermentation of *Zanthoxylum* seed oil meal with waste bacterial bran and biocontrol bacteria significantly improved nutrient conversion and microbial activity in compost, reduced the abundance of antibiotic resistance genes, and had a better control effect on tobacco root disease. However, the effects of cow manure mixed with botanical oil meal on the soil microbial community structure and function as well as tobacco quality in tobacco fields are still unclear.

There are significant differences in the macroelements contained in different manures. The single application of cow manure leads to the imbalance of essential elements required by soil, and its ability to rapidly improve soil health status is insufficient (Su et al., 2022). The nutrient content of organic fertilizer obtained from plants is not rich, the supplementation of soil nutrients is insufficient, and the effect is relatively slow. However, the improvement effect of organic fertilizer on soil quality is obvious, especially for soil with severe compaction, salinization, and acidification (Zhao et al., 2022). In this study, soybean, rapeseed, peanut, and sesame meals were composted with cow manure and then applied to tobacco field soil to study the changes in the soil microbial community structure, function, and effects of soil physicochemical properties and enzyme activities on soil microorganisms, further evaluating the effects of application on the growth and quality of tobacco plants. The results have important implications for the evaluation of the soil microecological effects of cow manure and botanical oil meal organic fertilizer and its application in tobacco.

## 2. Materials and methods

### 2.1. Mixed compost of cow manure and botanical oil meal

The cow manure was dried and drained, the moisture content of which was controlled to below 80%, and then different botanical oil meals were added (soybean meal, rape meal, peanut bran, and sesame meal). The ratio of cow manure to different plant oil meals was 7:3 (mass ratio), and a small amount of organic fertilizer fermentation Effective Microorganisms bacteria was added. The water content was controlled at 70%, the compost was mixed and stirred evenly, pile fermentation, until the temperature reached above 60°C, and was mixed after 48 h. After seven days for one pile and four times of piling, the cow manure and botanical oil meal were fully decomposed and the cow manure–botanical oil meal organic fertilizer was prepared.

### 2.2. Field experiment design

The test area was located in the tobacco-producing area of Jingxi City, Guangxi Province (105°56’E, 22°51’N). A single-factor completely random block design was used for a total of six treatments (CK: no fertilization; NF: administration of 1500 kg/hm^2^ of cow manure; NFC: application of 1500 kg/hm^2^ of cow manure and rapeseed bran organic fertilizer; NFD: application of 1500 kg/hm^2^ of cow manure and soyabean meal organic fertilizer; NFHS: application of 1,500 kg/hm^2^ of cow manure and peanut bran organic fertilizer; NFZM: application of 1,500 kg/hm^2^ of cow manure and sesame meal organic fertilizer). The main nutrient contents of various organic fertilizers are shown in Supplementary Table 1. Each process had three replicates for a total of 18 blocks. Before transplanting, the fertilizer was used as the base fertilizer. After the fertilizer was spread, the land was ploughed to mix the fertilizer evenly with the soil, and then the K326 tobacco seedlings with vigorous growth and developed root systems were transplanted to the field. At the time of tobacco harvesting, 10 representative tobacco plants were randomly taken from each plot, and the plant height, leaf number, maximum leaf length, and maximum leaf width were measured according to the tobacco agronomic trait survey method (YC/T 142-1998). The tobacco leaves were harvested, then baked in the baking room, and the flue-cured tobacco yield was calculated after the baking was completed. The tobacco was graded according to GB/T5606.1-2004.

### 2.3. Soil sample collection and determination of physicochemical properties

Two months after tobacco transplanting, each treatment was sampled using a five-point sampling method. First, the soil surface leaves, gravel, and other debris were swept away, and then soil samples at 5–20 cm were collected using a soil drill with a diameter of 3.5 cm, and about 2 kg of soil was taken from each community. Each soil sample was divided into two parts. One part (0.5 kg) was stored at −80°C for soil microbial analysis and the other part (1.5 kg) was used for the analysis of soil physicochemical properties and the detection of soil enzyme activity.

Soil ammonium nitrogen and nitrate nitrogen were extracted using a potassium chloride solution. Ammonium nitrogen can react with phenol to form blue indigo phenol dye, nitrate nitrogen can react with hydrochloride *N*(1-naphthyl)-ethylenediamine to form red dye, and the two have maximum absorption at 630 and 543 nm, respectively. This method can be used to determine the content of soil ammonium nitrogen and nitrate nitrogen (Page, 1983). The available phosphorus content in soil was extracted using sodium bicarbonate extractant, and the content of available phosphorus was measured using the continuous flow analysis of air fragments (Olsen, 1954). Soil available potassium was leached in a neutral 1 mol/L ammonium acetate solution and determined using a flame photometer (Model 410. Sherwood scientific Ltd Cambridge, UK). The determination of soil organic matter content followed the potassium dichromate oxidation-volumetric method (Franz et al., 1996). Soil pH was measured using a pH meter (China Shanghai Sanxin Instrument Co., Ltd.) in a 1:2.5 (w/v) soil solution. Electrical conductivity (EC) was measured with a 1:2.5 soil/water suspension using an MP513 conductivity meter (Shanghai Sanxin Instrument Co., Ltd., China). Ten grams of fresh soil was taken and dried at 105°C for 12 h, and then the soil moisture content was measured using the drying weighing difference method.

### 2.4. Determination of soil enzyme activity

Soil alkaline phosphatase (S-AKP) activity was determined via diasodium phosphate colorimetric chromometry using the Soil Alkaline Phosphatase Activity Assay Kit (Liang et al., 2017). Soil catalase activity (S-CAT) was determined based on ultraviolet absorption using the Soil Catalase Activity Assay Kit (Geng et al., 2017). Soil peroxidase activity (S-POD) was determined by catalyzing the oxidation of organic matter to quinone using the Soil Peroxidase Activity Assay Kit (Tian and Shi, 2014). The absorbance values of alkaline phosphatase (AKP), catalase (CAT), and peroxidase (POD) at 660, 240, and 430 nm, respectively, were measured using an ultraviolet-visible spectrophotometer (UV-5100B) to reflect their respective enzyme activities. All kits were purchased from Beijing Box Shenggong Technology Co., Ltd., and the ultraviolet-visible spectrophotometer was produced by Shanghai Yuanxi Instrument Co., Ltd.

### 2.5. Soil DNA extraction and high-throughput sequencing

Total genomic DNA was extracted from each soil sample using the DNeasy PowerSoil Kit (100) isolation kit (QIAGEN Co. Ltd., Germany). The quality and concentration of extracted DNA were determined using 2% agarose gel electrophoresis and nanodroplets NanoPhotometer N50 Touch (Implen Gmbh Co. Ltd., Germany).

The V3–V4 region of the bacterial 16SrRNA gene was amplified using the universal primers 338F (5′-ACTCCTACGGGAGCAGCAGCAG-3′) and 806R (5′-GGACTACHVGGGTWTCTAAT-3′) (Nan et al., 2016). The internal transcribed spacer (ITS) of the fungus was amplified using the universal primers ITS1F (5′-CTTGGTCATTTAGAGGAAGTAA-3′) and ITS2R (5′-GCTGCGTTCTCCATCGATGC-3′) (Rachel et al., 2013). The PCR reaction consisted of 2 μL 10 × buffer, 2 μL 2.5 mM dNTPs, 0.8 μL forward primer (5 μM), 0.8 μL reverse primer (5 μM), 0.4 μL polymerase, 0.2 μL Bovine serum albumin, 10 ng template DNA, and finally the addition of ddH_2_O to reach a total volume of 20 μL. The procedure for the PCR reaction was as follows: predenaturation at 95°C for 3 min; then denaturation at 95°C for 30 s and annealing at 55°C for 30 s; followed by extension for 45 s at 72°C, and these three steps were repeated for a total of 35 cycles; followed by single extension at 72°C for 10 min; and ending at 4°C. The PCR products were sequenced on the Illumina MiSeq platform of Majorbio Bio-Pharm Technology Co., Ltd. (Shanghai, China).

### 2.6. Bioinformatics analysis and data processing

The off-machine raw data was spliced using FLASH (v1.2.11). QIIME (Quantitative Insights Into Microbial Ecology, v1.9.1) was used for quality control of the original sequences. Using Uparse (v11), the obtained high-quality sequences were clustered into operational taxonomic units (OTUs) ≥ a similarity threshold of 97% (Edgar et al., 2011). The α diversity of fungal communities was analyzed based on the Chao and Shannon indices using the Mothur (v.1.30.0) software (Schloss et al., 2009). Redundancy analysis (RDA) was performed using the R (v.3.3.1) software package “Vegan,” and primary coordinate analysis (PCoA) was performed via direct mapping based on Euclidean distances. Python (v.2.7.0) software was used to analyze the relative abundances of species. Linear discriminant analysis effect size (LEFSe) analysis was used to linearly discriminate samples according to different classification methods (Segata et al., 2011). Using linear discriminant analysis (LDA), species with significant differences were found in the sample classification. The OTU abundance table of soil bacteria was normalized using PICRUSt1, and then Kyoto Encyclopedia of Genes and Genomes (KEGG) function-annotated OTUs based on the greengene ID corresponding to each OTU. According to FUNGuild, the functional classification of soil fungi and the abundance information of each functional classification in different samples were obtained. The pheatmappackage and R vegan package were used to generate heatmaps and statistical correlations (Kolde, 2015). Statistical analysis was conducted using IMB SPSS Statistics 25*^th^* Edition (IBM, USA) and supplementary calculations were performed in Microsoft Excel 2016. Using Duncan’s new complex range method, one-way analysis of variance (ANOVA) was used to analyze changes in soil physicochemical properties and enzyme activity. R software was used to plot a visualization of environmental factor correlation analysis. OriginPro^®^2018 (OriginLab Corp., USA) and Adobe Illustrator CC2019 (Adobe Systems Inc., USA) software were used for drawing.

## 3. Results and analysis

### 3.1. Changes in tobacco growth indicators, yield, and quality

Supplementary Table 2 shows the effects of the single application of cow manure and the mixed application of cow manure with different botanical oil meals on tobacco growth indicators. For the maximum blade width, the NFHS treatment was the largest and significantly higher compared to the NF, NFD, and NFZM treatments. While the NF treatment maximum blade width was the smallest, there was no significant difference between it and NFC, NFD, and NFZM treatment.

Supplementary Table 3 shows the effects of the single application of cow manure and the mixed application of cow manure with different botanical oil meals on the yield and quality of tobacco. The results showed that the mixed application of cow manure and different botanical oil meals could increase the yield of tobacco, and the largest yield was found in the NFHS treatment (NFC: 14.29%; NFD: 11.94%; NFHS: 28.29%; and NFZM: 21.34%). All fertilization treatments could increase the proportion of fine tobacco, and the largest proportion was found in the NFD treatment (NFC: 20.03%; NFD: 41.21%; NFHS: 10.24%; and NFZM: 34.53%). There was no significant difference in average price among all treatments, and the largest difference was 1.82 yuan/kg in the NFC treatment. The NFHS treatment had the highest output value, mainly because its output was much higher than those of the other treatments.

### 3.2. Changes in soil physicochemical properties

Compared with CK, the content of NH_4_^+^-N in the soil in the NF treatment was the highest, and significantly higher than that in the NFC and NFD treatments. The content of soil NO_3_^–^-N was the highest in the NFHS treatment and the lowest in the NFZM treatment, and there were significant differences between the two and the CK, NF, and NFD treatments. The soil available-P content in the NFD and NFHS treatments was the highest, which was significantly higher than that in the CK and other treatments. The content of soil available-K was the highest in the NFD treatment, and there were significant differences among all fertilization treatments except for the NF and NFZM treatments. The soil organic matter content in CK was the lowest and was significantly lower than that in the NFC, NFD, NFHS, and NFZM treatments, but there was no significant difference in soil organic matter content among the four treatments. The soil pH content in the NFZM treatment was the highest, and there was no significant difference between the other four treatments and the control treatment, which were all significantly lower than that in the NFZM treatment. The EC of NFC and NFD was the highest and was significantly higher than that of the other four treatments, and the EC of the NFZM treatment was the lowest. Compared with the NF treatment, the available-K and EC of the NFC treatment were significantly increased; the available-K, available-P, and EC in the NFD treatment were significantly increased; and the available-P, available-K, and NO_3_^–^-N were significantly increased in the NFHS treatment. The pH of the NFZM treatment was significantly increased (Table 1).

**TABLE 1:** Physicochemical properties of soil under different treatments.

Treatment	NH_4_^+^-N (mg/kg)	NO_3_^–^-N (mg/kg)	Available-P (mg/kg)	Available-K (mg/kg)	Organic matter(mg/kg)	pH (1:2.5)	EC (μs/cm)
CK	0.35 ± 0.07ab	35.73 ± 2.16b	112.77 ± 2.49b	386.00 ± 10.55e	43.10 ± 1.78b	7.41 ± 0.03b	508.00 ± 10.02bc
NF	0.55 ± 0.09a	35.73 ± 0.18b	118.30 ± 2.52b	465.83 ± 7.95d	50.90 ± 3.89ab	7.50 ± 0.06b	487.00 ± 22.65bc
NFC	0.44 ± 0.05ab	40.83 ± 2.12ab	122.77 ± 3.96b	674.17 ± 41.84c	53.43 ± 2.97a	7.47 ± 0.03b	842.67 ± 59.37a
NFD	0.46 ± 0.09ab	36.73 ± 2.82b	182.67 ± 12.2	915.83 ± 15.90a	52.83 ± 2.33a	7.47 ± 0.01b	833.00 ± 34.04a
NFHS	0.27 ± 0.08b	46.23 ± 1.66a	a170.60 ± 6.68a	736.67 ± 8.21b	53.43 ± 3.77a	7.41 ± 0.02b	604.33 ± 57.32b
NFZM	0.39 ± 0.04b	24.57 ± 0.61c	117.17 ± 6.25b	461.67 ± 7.12d	52.80 ± 2.15a	7.61 ± 0.02a	390.67 ± 9.77c

The letters “a, b, c and d” are significant markers, and different letters between treatments indicate significant differences at the significant level of 0.05.

### 3.3. Changes in soil enzyme activity

**FIGURE 1:**
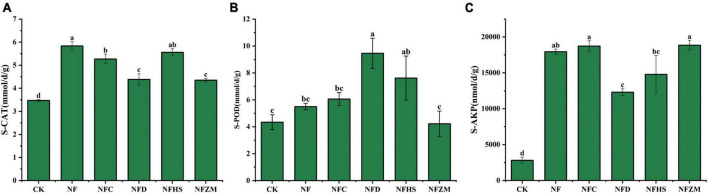
Activities of three soil enzymes under different treatments. **(A)** Soil catalase activity; **(B)** soil peroxidase activity; **(C)** soil alkaline phosphatase activity.

Compared with the CK, although there was no significant difference between the S-POD activities of NFZM and CK, the other treatments increased the activities of S-CAT, S-POD, and S-AKP. In general, the improvement of S-AKP activities was the largest (NF: 5.93 times; NFC: 5.67 times; NFD: 3.38 times; NFHS: 4.27 times; and NFZM: 5.71 times). Compared with cow manure alone, the NFC, NFD, and NFZM treatments significantly reduced the activity of S-CAT (Figure 1A); the NFD treatment significantly increased the activity of S-POD (Figure 1B); and the NFD treatment significantly reduced the activity of S-AKP (Figure 1C).

### 3.4. Changes in soil microbial diversity

**FIGURE 2:**
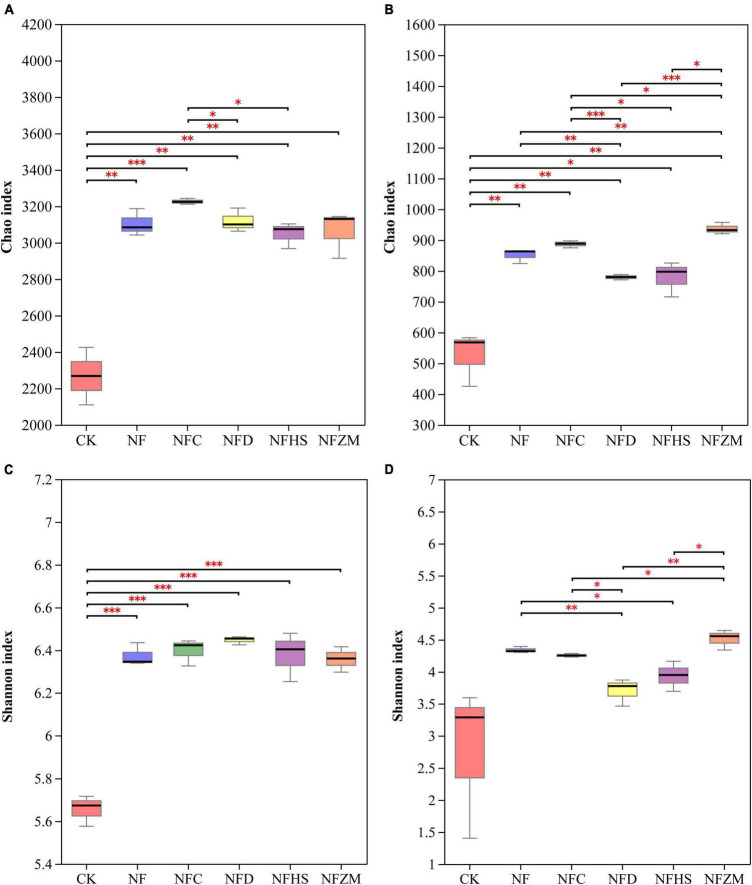
α diversity index of soil microorganisms. **(A)** Chao index of soil bacterial community; **(B)** Chao index of soil fungal community; **(C)** Shannon index of soil bacterial community; **(D)** Shannon index of soil fungal community. An asterisk represents the difference at the 0.05 level, meaning the difference is significant. The two asterisks represent a 0.01 level difference, meaning the difference is very significant. The three asterisks represent the 0.001 level, meaning the difference is extremely significant.

In the soil bacterial community, compared with CK, the Chao index of each fertilization treatment was extremely significantly higher than CK, and the increase in the NFC treatment was the most obvious (Figure 2A). The Shannon index values of soil bacteria in each fertilization treatment were significantly higher than that of CK (Figure 2C). In the soil fungal community, compared with CK, the Chao index of each fertilization treatment was extremely significantly higher than that of CK (Figure 2B). However, in the Shannon index of soil fungi, there was no significant difference between each fertilization treatment and CK (Figure 2D). By comparing the NF treatment with the fertilizer treatment with the addition of different botanical oil meals, it was found that there were no significant differences in the Chao index and Shannon index between the NF treatment and the NFC, NFD, NFHS, and NFZM treatments in soil bacterial communities. In the soil fungal community, compared with NF treatment, the Chao index of the NFZM treatment was significantly increased, the Shannon index of the NFHS treatment was significantly decreased, and both the Chao index and Shannon index of the NFD treatment were significantly decreased.

**FIGURE 3:**
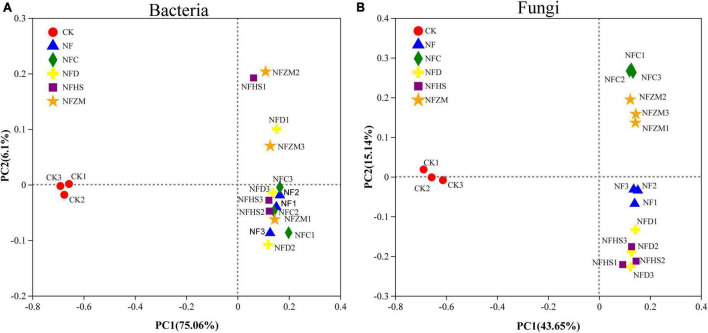
Variation of soil microbial β diversity. **(A)** PCoA analysis of soil bacterial communities. **(B)** PCoA analysis of soil fungal communities.

The results of PCoA showed that no treatment (CK) and the single application of cow manure (NF), cow manure combined with rapeseed meal (NFC), cow manure combined with soybean meal (NFD), cow manure combined with peanut meal (NFHS), and cow manure combined with sesame meal (NFZM) could be significantly separated based on the PC1 axis. The contribution rates of PC1 and PC2 to the species composition difference between treatments were 75.06%/6.1% (bacteria) and 43.65%/15.14% (fungi), respectively (Figures 3A, B). In the soil fungal community, the fertilization treatments and CK could be significantly distinguished on the PC1 axis. The fertilization treatments, although were close on the PC1 axis, could be distinguished on the PC2 axis; this indicated that the application of cow manure and cow manure mixed with different botanical oil meals could change the soil fungal community structure. In the soil bacterial community, CK and each fertilization treatment could be significantly distinguished by the PC1 axis. Different from fungal communities, the NF and NFC treatments of soil bacterial communities were similar on the PC2 axis, indicating that soil bacterial communities applied with cow manure and cow manure mixed with botanical oil meal were similar.

### 3.5. Analysis of soil microbial composition

**FIGURE 4:**
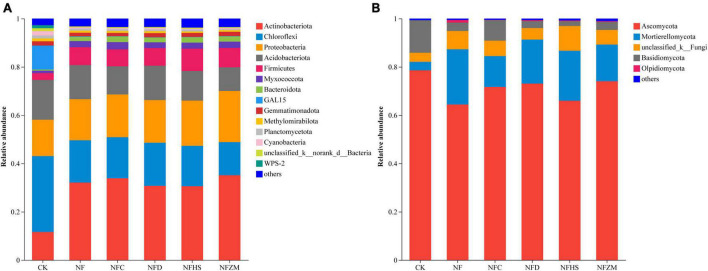
Analysis of soil microbial composition. **(A)** Soil bacterial community. **(B)** Soil fungal community.

Figure 4 shows the composition in soil microorganisms at the phylum level under different treatments. In the soil bacterial community, the top five relative abundance were Actinobacteriota (11.65, 32.07, 33.88, 30.72, 30.61, and 35.17% in CK, NF, NFC, NFD, NFHS, and NFZM, respectively); Chloroflexi (31.44, 17.55, 17.03, 17.86, 16.71, and 13.68%, respectively); Proteobacteria (15.05, 17.00, 17.70, 17.68, 18.71, and 21.16%, respectively); Acidobacteriota (16.48, 14.13, 11.63, 14.21, 12.29, and 9.84%, respectively); and Firmicutes (2.80, 7.42, 7.03, 7.29, 9.25, and 7.97, respectively). Notably, the relative abundance of GAL15 in CK was much higher than that in other treatments (9.93%), which was 486.5–983% higher than that in other treatments (Figure 4A). In the soil fungal community, the dominant phyla with the highest relative abundances were Ascomycota (the relative abundances in CK, NF, NFC, NFD, NFHS, and NFZM were 78.60, 64.44, 71.69, 73.08, 65.98, and 74.08%, respectively); Mortierellomycota (3.49, 22.87, 12.81, 18.24, 20.71, and 15.15%, respectively); Basidiomycota (13.49, 3.47, 8.52, 2.91, 2.02, and 3.32%, respectively); and Olpidiomycota (0, 1.06, 0.11, 0.36, 0.26, and 0.33%, respectively). There was a small amount of accumulation in all treatments, but not in CK (Figure 4B).

### 3.6. Analysis of different species of soil microorganisms

According to the results of the effects of different treatments on the soil physicochemical properties and tobacco yield and quality, the NFD, NFHS, and NFZM treatments as well as the NF treatment were selected for LEFSe multilevel species difference analysis. The classification level ranged from phylum to genus, and the LDA threshold was 3.

**FIGURE 5:**
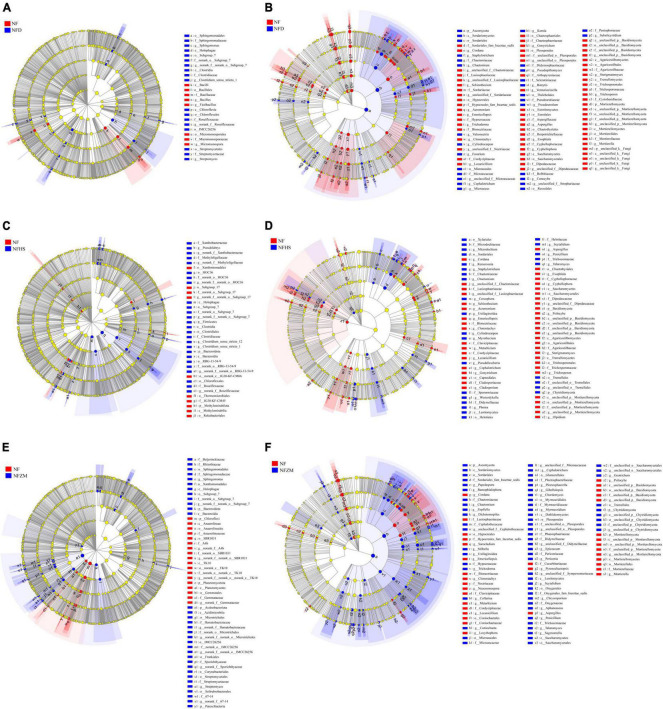
Analysis of multilevel species differences of soil microorganisms under different treatments. **(A)** Analysis of multilevel species differences between NF and NFD soil bacterial communities. **(B)** Multilevel species differences between NF and NFD soil fungal communities. **(C)** Analysis of multilevel species differences between NF and NFHS soil bacterial communities. **(D)** Multilevel species differences between NF and NFHS soil fungal communities. **(E)** Multilevel species differences between NF and NFZM soil bacterial communities. **(F)** Multilevel species differences between NF and NFZM soil fungal communities.

In the soil bacterial community, *Bacillus*, *Fictibacillus*, and *Micromonosporaceae* were significantly enriched in the NF treatment compared with the NFD treatment, while *Sphingononas* and *Streptomyces* were significantly enriched in the NFD treatment (Figure 5A). Compared with the NFHS treatment, at the phylum level, Methylomirabilota was significantly enriched in the NF treatment, while Firmicutes, Bacteroidota, and *Pseudolabrys* were significantly enriched in the NFHS treatment (Figure 5C). Compared with the NFZM treatment, at the phylum level Chloroflexi and Planctomycetota were significantly enriched in the NF treatment group, while the phyla Bacteroidota and Actinobacteriota as well as the genera *Spingononas* and *Streptomyces* were significantly enriched in the NFZM treatment (Figure 5E). In the soil fungal community, compared with the NFD treatment, Mortierellomycota at the phylum level and *Cordana*, *Schizothecium*, *Venturiocistella*, *Sterigmatomyces*, and *Trichosporon* at the genus level were significantly enriched in the NF treatment. The phylum Ascomycota and the genera *Conocybe* and *Subulecystidium* were significantly enriched in the NFD treatment (Figure 5B). Compared with the NFHS treatment, the Chytridiomycota phylum and the *Schizothecium*, *Acremonium*, *Basidiomycota Emericellopsis*, *Metarhizium*, *Lecanicillium*, *Cephalotrichum*, *Gonytrichum*, *Gonytrichum*, *Aspergillus*, *Psilocybe*, and *Olpidium* genera were significantly enriched in the NF treatment. The Chytridiomycota phylum and the *Ustilaginoidea*, *Myrothecium*, *Westerdykella*, *Phoma*, *Penicillum*, and *Talaromyces* genera were significantly enriched in the NFHS treatment (Figure 5D). Compared with the NFZM treatment, the Mortierellomycota phylum and the *Cordana*, *Sanocladium*, *Emerlopsis*, *Clonostachys*, *Neocosmospora*, *Metarhizium*, *Lecythophora*, *Pyrenochaetopsis*, *Aspergillus*, and *Psilocybe* genera were significantly enriched in the NF treatment, and the Ascomycota and Chytridiomycota phyla, as well as the *Chaetomium*, *Zopfiella*, *Dichotomopilus*, *Gibellulopsis*, and *Penicillium* genera, were significantly enriched in the NFZM treatment (Figure 5F).

### 3.7. Correlation analysis of soil physicochemical properties and enzyme activities with microorganisms

**FIGURE 6:**
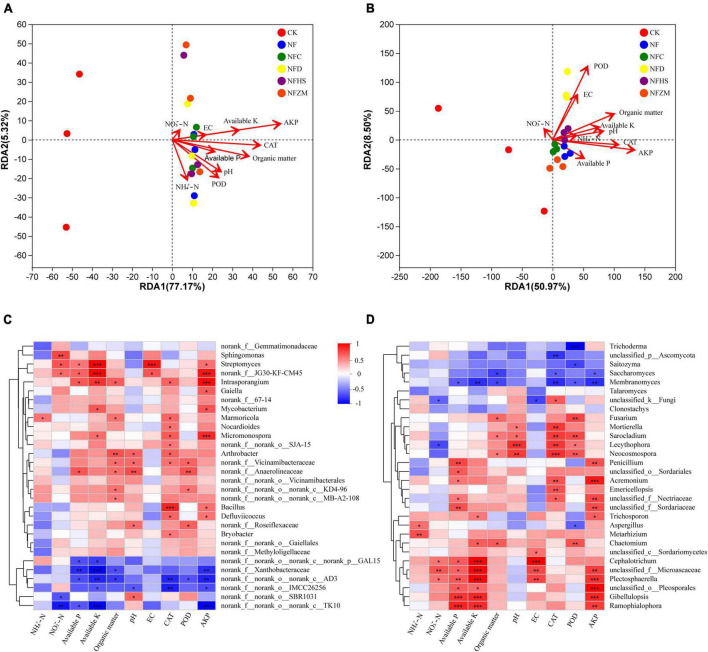
Correlation analysis of soil physicochemical properties and enzyme activities with microorganisms. **(A)** RDA analysis of soil physicochemical properties, enzyme activities and soil bacteria; **(B)** RDA analysis of soil physicochemical properties, enzyme activities and soil fungi; **(C)** Heatmap analysis of correlation between soil physicochemical properties, enzyme activities and soil bacteria; **(D)** Heatmap analysis of correlation between soil physicochemical properties, enzyme activities and soil fungi. An asterisk represents the difference at the 0.05 level, meaning the difference is significant. The two asterisks represent a 0.01 level difference, meaning the difference is very significant. The three asterisks represent the 0.001 level, meaning the difference is extremely significant.

This work studied the relationship between the soil physicochemical properties, enzyme activities, and microbial community on the genus level using RDA analysis. The results showed that the interpretative values of the RDA1 and RDA2 axes for soil bacteria and fungi were 77.17%/5.32% and 50.97%/8.50%, respectively (Figures 6A, B). In the soil bacterial community, AKP had the greatest influence on the soil bacterial community, while NO_3_^–^-N had the least. Except for the negative correlation between NH_4_^+^-N and NO_3_^–^-N, other soil physicochemical properties and enzyme activities were positively correlated. Overall, the microorganisms in the NF, NFC, NFD, and NFHS treatments were positively correlated with soil physicochemical properties and enzyme activities. The NFZM treatment was negatively correlated with NH_4_^+^-N and positively correlated with other soil physicochemical properties and enzyme activities. In the soil fungal community, AKP had the greatest influence on soil fungi, while NO_3_^–^-N had the least influence. Among all soil physicochemical properties and enzyme activities, NO_3_^–^-N was only positively correlated with POD and EC, available-P was only negatively correlated with NO_3_^–^-N, POD, and EC, and other soil physicochemical properties were positively correlated with enzyme activities. In each fertilization treatment, CK was positively correlated with NO_3_^–^-N, while the soil fungal communities in the NFC, NFD, NFHS, and NFZM treatments were positively correlated with available-P, AKP, CAT, NH_4_^+^-N, PH, available-K, and organic matter, and negatively correlated with EC, POD, and NO_3_^–^-N.

This study assessed the correlations between soil physicochemical properties, enzyme activities, and microorganism using correlation heatmaps (Figures 6C, D), which showed the top 30 species in total abundance at the genus level. In the soil bacterial community, *Intrasporangium*, *Micromonospora*, *Streptomyces*, *Gaiella*, *Mycobacterium*, *Bacillus*, and *Defluviicoccus* showed significant positive correlations with AKP, while *norank_f_norank_o_norank_c_TK10*, *norank_f_norank_o_norank_c_AD3*, *norank_f_Xanthobacteraceae*, and *norank_f_norank_o_IMCC26256* showed significant negative correlations with AKP. In addition, it was found that the correlation heatmap could further explain the results of the RDA analysis. AKP, available-K, and EC were in a straight line in the RDA analysis, and their colors were similar in the correlation heatmap. However, because EC had the shortest length in the RDA analysis, its significance in the correlation heat map was not as obvious as those of AKP and available-K. In the soil fungal community, *Acremonium*, *Plectosphaerella*, *Gibellulopsis*, *Penicillium*, *Ramophialophora*, and *Trichosporon* showed significant positive correlations with AKP, while *Membranomyces* and *Saccharomyces* showed significant negative correlations with AKP. It was worth noting that *Plectosphaerella* was significantly positively correlated with five soil physicochemical properties and enzyme activities, while *Membranomyces* was significantly negatively correlated with six soil physicochemical properties and enzyme activities, which are key components of the soil fungal community.

### 3.8. Prediction and analysis of soil microbial community function

**FIGURE 7:**
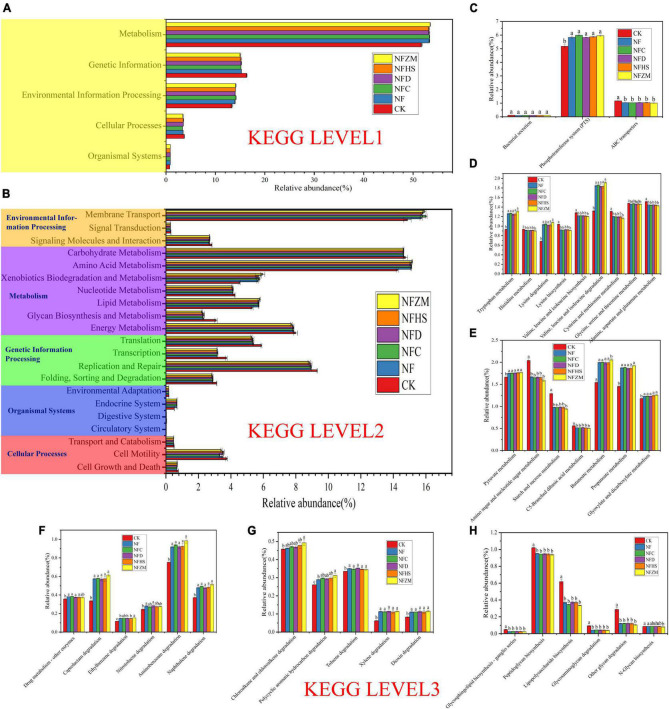
KEGG prediction analysis of functional genes of metabolic pathways in soil bacteria. **(A)** Prediction of soil bacterial metabolic pathway at Level 1; **(B)** Prediction of soil bacterial metabolic pathway at Level 2 level; **(C)** Function prediction of membrane transport at Level 3; **(D)** Functional prediction of amino acid metabolism Level 3; **(E)** Functional prediction of carbohydrate metabolism Level 3; **(F,G)** Function prediction of xenobiotics biodegradation and metabolism at Level 3; **(H)** Functional prediction of glycan biosynthesis and metabolism at Level 3.

The functions of soil bacterial communities in KEGG metabolic pathways were analyzed using PICRUSt prediction. The five functions related to microbial metabolism in KEGG were metabolism (51.70–53.46%), genetic information (15.03–16.36%), environmental information processing (13.35–14.17%), cellular processes (3.33–3.72%), and organismal systems (0.74–0.87%) (Figure 7A). At KEGG level 2, the top three functions were membrane transport (14.87–16.02%), amino acid metabolism (14.25–15.16%), and carbohydrate metabolism (14.61–14.74%) (Figure 7B). Xenobiotics biodegradation and metabolism as well as glycan biosynthesis and metabolism were also worth attention. Compared with CK, the increase (24.32–31.32%) and decrease (24.84–28.52%) of the two fertilization treatment treatments were the largest. Therefore, at KEGG Level 3, this work focused on the changes of specific metabolic pathway functional genes involved in membrane transport, amino acid metabolism, carbohydrate metabolism, xenobiotics biodegradation and metabolism, and glycan biosynthesis and metabolism.

In membrane transport (Figure 7C), the abundance of functional genes of the phosphotransferase system (PTS) was the highest. Compared with the CK, the NF, NFC, NFD, NFHS, NFZM treatments were significantly higher. For the functional genes of ABC transports, those in the fertilization treatments were significantly lower than those in CK, while there was no significant difference in bacterial secretion between the fertilization treatments and CK. For amino acid metabolism (Figure 7D), compared with CK, the relative abundances of functional genes related to tryptophan metabolism, lysine metabolism and valine, leucine, and isoleucine degradation were significantly increased in all treatments, among which the NFZM treatment was the highest, but there was no significant difference between treatments. Compared with CK, the relative abundances of functional genes in histidine metabolism; lysine biosynthesis; valine, leucine, and isoleucine biosynthesis; cysteine and methionine metabolism; and alanine, aspartate, and glutamate metabolism were significantly decreased in all treatments, and there was no significant difference between treatments. In carbohydrate metabolism (Figure 7E), compared with CK, the relative abundances of functional genes in pyruvate metabolism, butanoate metabolism, propanoate metabolism, and glyoxylate and dicarboxylate metabolism in all treatments were significantly increased, and there was no significant difference between treatments. However, the relative abundances of functional genes in amino sugar and nucleotide sugar metabolism, starch and sucrose metabolism, and C5-branched dibasic acid metabolism were significantly decreased, and there was no significant difference between treatments. In xenobiotics biodegradation and metabolism (Figures 7F, G), the relative abundances of functional genes of the metabolic pathways in each fertilization treatment were significantly higher compared to CK, among which the relative abundance of the functional genes of the top five metabolic pathways were aminobenzoate degradation, caprolactam degradation, naphthalene degradation, chloroalkane and chloroalkene degradation, and toluene degradation. Importantly, compared with CK, the relative abundances of functional genes for ethylbenzene degradation and polycyclic aromatic hydrocarbon degradation in the NFZM treatment were significantly increased. In glycan biosynthesis and metabolism (Figure 7H), compared with CK, the relative abundances of functional genes of peptidoglycan biosynthesis, lipopolysaccharide biosynthesis, other glycans degradation, glycosphingolipid biosynthesis-ganglio series, and glycosaminoglycan degradation were significantly decreased. For N-glycan biosynthesis, only the NFZM treatment was significantly decreased compared with CK.

**FIGURE 8:**
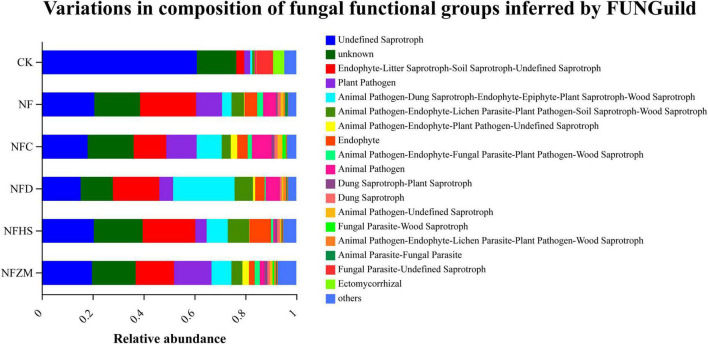
Predictive analysis of functional genes of soil fungal metabolic pathways.

FUNGuild was used to predict the function of soil fungal communities (Figure 8). Regardless of CK or each fertilization treatment, the relative abundances of undefined saprotrophs, endophyte- litter saprotroph-soil saprotroph-undefined saprotrophs, and plant pathogens accounted for more than 50%, indicating that they constituted the majority of the soil fungal community. Compared with CK, the relative abundances of undefined saprotrophs in each fertilization treatment decreased, while the relative abundances of endophyte- litter saprotroph-soil saprotroph-undefined saprotrophs increased. Notably, there were different functional communities in each treatment. The relative abundances of animal pathogens in the NFC treatment was higher than that in the other groups. In the NFD treatment, the relative abundance of animal pathogen-dung saprotroph-endophyte-epiphyte-plant saprotroph-wood saprotrophs was higher than that in other treatments. In the NFHS treatment, the relative abundance of animal pathogen-endophyte-lichen parasite-plant pathogen-wood saprotrophs was higher than that in other treatments. The relative abundance of plant pathogens in the NFZM treatment was higher than that in other treatments.

## 4. Discussion

### 4.1. Effects of the mixed application of cow manure and botanical oil meal on the growth, yield, and quality of tobacco

The crop growth, yield, and quality are the most direct reflections of the crop growth environment. The results showed that compared with other fertilization treatments, the combined application of cow manure and peanut bran had the highest tobacco agronomic traits and yield. Compared with other organic fertilizers, peanut bran had a lower carbon: nitrogen ratio, a faster decomposition rate after application in soil, and a faster fertilizer effect. This treatment could provide full nutrition to tobacco in the seedling stage, which helped the roots absorb the nutrients more quickly. The nutrients then spread to all parts of the plant. The findings of this study were consistent with previous results. Roeswitawati et al. (2021) found that peanut bran and biological mud had significant effects on the growth and yield of sweet corn, with 15 tons/ha of peanut bran and 125 ml/L of biological mud having the best effect. The average price of flue-cured tobacco was the highest after the mixed application of cow manure and rapeseed oil meal, which indicated that rapeseed oil meal could improve the quality of flue-cured tobacco.

### 4.2. Effects of the mixed application of cow manure and botanical oil meal on soil physicochemical properties

The physicochemical properties of soil are the key factors that determine soil fertility. The long-term application of chemical fertilizers or the continuous application of the same manure would cause the unpredictable consumption of soil nutrients, which seriously damages soil productivity. The results showed that the mixed application of cow manure with different botanical oil meals had different changes in soil available-P, available-K, EC, NO_3_^–^-N, and pH: rapeseed oil meal significantly increased the effect of EC, soybean meal significantly increased the effect of available-P and available-K, peanut bran significantly increased the effect of NO_3_^–^-N, and sesame meal significantly increased the effect of pH. The soil available-P, available-K, EC, NO_3_^–^-N, and pH interacted with each other to some extent. Soil with neutral pH can reduce the fixation effect of P and improve the availability of soil P (Muyang et al., 2016). For soil with high organic matter content, the mineralization of organic matter can provide part of the required inorganic phosphorus, which weakens the effect of fixing phosphorus and increases the content of soil available-P. The NFHS treatment had the lowest NH_4_^+^-N content and the highest NO_3_^–^-N content. NH_4_^+^-N and NO_3_^–^-N are converted into each other through nitrification and denitrification. NO_3_^–^-N is easily dissolved and has high activity in the soil, which can quickly provide nitrogen nutrition for crops; however, it is also easy to lose. This characteristic meets the growth demand for tobacco; tobacco grows rapidly and can turn senescence and mature at an appropriate time (Ghiasi et al., 2021).

### 4.3. Effects of the mixed application of cow manure and botanical oil meal on soil enzyme activities

Soil enzyme activity reflects the ability of soil to perform catalytic substance conversion. The activity mainly derived from the secretions of soil animals, plants, and microorganisms as well as the decomposition of various residues. To some extent, soil enzyme activity participate in the occurrence and development of soil and the formation and evolution of soil fertility (Li et al., 2023). Yang et al. (2022) found that deep tillage accompanied by the application of organic fertilizer increased the activities of sucrase, cellulase, and urease in wheat fields; furthermore, Liu et al. showed that the application of organic fertilizer, chemical fertilizer (nitrogen fertilizer), and organic fertilizer combined with chemical fertilizer increased the activities of sucrase in soil by 124.1, 80.9, and 145.6%, respectively, while urease and alkaline phosphatase were also increased to varying degrees (Liu et al., 2021). These findings were consistent with the results of the present study. The application of cow manure and mixed application of cow manure and botanical oil meal could improve soil enzyme activity. The effects of adding different botanical oil meals on soil enzyme activity were different. After the addition of soybean meal, S-POD activity was significantly increased compared with cow manure applied alone, but S-AKP activity was significantly decreased. This might be because the N element in the soybean meal was higher than that in the other botanical oil meals, while the P element content was low. Therefore, soil microbial reactions related to the metabolism of N were enhanced, while the number of reactions associated with P decreased.

### 4.4. Effects of the mixed application of cow manure and botanical oil meal on soil microbial diversity

For soil bacterial communities, the Chao index and Shannon index were significantly increased by the single application of cow manure and the combined application of cow manure and botanical oil meal. For soil fungal communities, the Chao index was increased by the single application of cow manure and the combined application of cow manure and botanical oil meal. Thuy et al. found that buffalo manure compost and vermicompost had positive effects on the activity of culturable bacteria and the bacterial Shannon index in soil (Thuy et al., 2013). Lupwayi et al. (2005) found that the Shannon index of soil bacteria was not significantly affected by cow manure and pig manure in the first two years, but significantly increased in the third year, and these results were consistent with the present study. Compared with the single application of cow manure, the addition of different botanical oil meals had no effects on the Chao index and Shannon index of soil bacteria, but for soil fungi, the addition of soybean meal significantly reduced the Chao index and Shannon index. The β diversity of microbial communities in each treatment was significantly different from that in CK. However, compared with cow manure application alone, although there were differences with the addition of different botanical oil meals, the difference was not as large as that in CK, which was verified based on the results of soil microbial α diversity.

### 4.5. Effects of the mixed application of cow manure and botanical oil meal on soil microbial composition

In the soil bacterial community, Actinobacteriota, Chloroflexi, and Proteobacteria accounted for more than 50% of soil bacteria and were the dominant phyla. Actinobacteriota includes important saprophytic bacteria that can promote the decay of plant and animal remains in soil and play a role in the nitrogen cycle in soil (Hazarika et al., 2022). Chloroflexi is a class of photoautotrophic bacteria known for their special 3-HP fixed CO_2_, making these bacteria of great significance to the entire ecosystem as primary producers (Narsing et al., 2022). Proteobacteria is the largest and most diverse phylum of bacteria and is of extensive importance in terms of phylogeny, ecology, and pathogenicity (Feng L. et al., 2023). Ascomycota occupies the greatest abundance in the soil fungal community. Studies have found that Ascomycota exists in soils all over the world and contains the pioneer decomposer species, whose main function is to ensure soil ecological security (Eleonora et al., 2019). The present study found that the abundance of Mortierellomycota in NF, NFC, NFD, NFHS and NFZM treatments was greater than that in CK. Studies have found that as a saprophytic fungus, Mortierellomycota has a high content in soil rich in organic matter and is a key microorganism in soil carbon and nutrient conversion (Shi et al., 2020). This was also confirmed by the results of the present study, as both the single application of cow manure and the combined application of cow manure with botanical oil meal increased the soil organic matter content.

LEFSe analysis revealed the microbial differences between cow manure and soybean meal, peanut bran, and sesame meal mixed application, and the single application of cow manure. The results showed that the combined application of cow manure with soybean meal, peanut bran, and sesame meal could increase the relative abundances of functional bacteria in soil compared with the single application of cow manure, and this effect was more obvious in the soil fungal community. In the soil bacterial community, *subgroup_7* was enriched with the application of the three botanical oil meals. Wang et al. found that the relative abundance of *subgroup_7* was significantly positively correlated with the 1000-grain weight, grain number per panicle, and yield of rice, and was a functional flora related to crop yield (Wang et al., 2021). As a saprophytic bacterium, *Spingomonas paucimobilis UT26* can simultaneously degrade methyl parathion and γ-HCH (Lan et al., 2014). Kruczyńska et al. reported that *Bacteroidota* can become an important indicator of soil quality in the study of soil biodegradation because the decrease of the abundance of these beneficial microorganisms may be related to the decrease of soil quality and fertility, and ultimately affect crop yield (Kruczyńska et al., 2023). In the soil fungal community, the three kinds of botanical oil meal were enriched in *Chaetomium* and *Penicillium*. *Chaetomium* can be used as a biocontrol bacterium. One *Chaetomium* species, *C. globosum* Cg-6, has a 60% inhibition potential against *Rhizoctonia solani*, and can increase the tuber germination rate (94%) (Arunkumar et al., 2021). *Penicillium* is a kind of phosphorus solution fungus that can be used as a bacteriological agent mixed with phosphate fertilizer and can transform phosphorus that is difficult to be absorbed and utilized by plants into a form that can be absorbed and utilized. This function is indispensable for the material cycle of the soil ecosystem (Chen et al., 2023).

### 4.6. Correlation analysis of soil physicochemical properties, enzyme activities, and soil microorganisms

AKP is the most influential factor for both soil bacteria and soil fungi. The level of S-AKP activity directly affects the decomposition and transformation of organic phosphorus in soil and its bioavailability and is an indicator to evaluate the direction and intensity of soil phosphorus biotransformation (Hu et al., 2015). Studies have shown that adding 0.01–3.0 mg/kg cadmium significantly improves the activity of S-AKP, and the soil bacterial community also responds to the addition of cadmium (Han et al., 2019). Chen et al. (2019). found that the long-term application of nitrogen fertilizer and pig manure decreased the activity of S-AKP and the abundances of phoD genes. *Intrasporangium* and *Micromonospora* are the soil bacteria most closely related to AKP. Studies have shown that there are freezing protein-coding sequences in *Intrasporangium*, and this strain has strong adaptability to harsh environments (Thomas et al., 2018). Karthikeyan et al. (2022) found that *Micromonospora*, as a biological control agent, can inhibit Fusarium wilt and promote the growth of host plants when it is colonized in the rhizosphere of *Casuarina*. We found that *Acremonium* and *Gibellulopsis* were the soil fungi with the strongest correlation with AKP. Khan et al. (2021) isolated the endophytic fungus *Acremonium* sp. Ld-03 from the bulb of *Tripterygium wilfordii*. It was used against *Fusarium oxysporum*, *Botrytis cinerea*, *Botryosphaeria dothidea*, and *Fusarium fujikuroi*. *Acremonium* sp. Ld-03 has certain inhibitory effects, and its secondary metabolites contain indoleacetic acid, which has a significant growth-promoting effect on leek. Studies have shown that *CEF08111*, a species of *Gibellulopsis*, can effectively control cotton verticillium wilt and induce a defense response in cotton plants (Feng Z. et al., 2023).

### 4.7. Prediction and analysis of the functional genes of metabolic pathways in soil microorganisms

For the functional prediction of the soil bacterial community, the relative abundance of Metabolism at LEVEL 1 was the largest, accounting for more than 50%, and the top three relative abundances at LEVEL 2 were membrane transport, amino acid metabolism, and carbohydrate metab olism, which was consistent with previous results (Huang et al., 2021). In membrane transport, the relative abundance of the functional genes of the phosphotransferase system was the largest, and the fertilization treatments had significantly higher abundances than CK, which also confirmed the previous results. AKP had the strongest correlation with soil microorganisms. Amino acids are an important component of soil organic nitrogen that can be used by some bacteria in the soil as precursors to synthesize plant growth regulators in the process of physiological metabolism to stimulate or promote plant growth and development (Tan et al., 2022). Tryptophan is an important precursor substance for auxin synthesis in plants, and its enhanced metabolism can regulate crop root morphology and promote root growth (Tashkandi et al., 2022). Enhanced lysine metabolism can promote chlorophyll synthesis, regulate stomatal opening, and promote leaf photosynthesis, which is conducive to crop yield increase (Fitriana et al., 2022). Carbohydrates are the most active organic component in soil, as well as one of the easily degraded components. There are interactions between carbohydrates, clay, and various ions that can stabilize the soil structure, and they also has the certain instruction to the change of soil microorganisms. In carbohydrate metabolism, pyruvate plays an important pivotal role by connecting glucose, fatty acid, and amino acid metabolism through acetyl CoA. Studies have shown that pyruvate metabolism is enhanced in non-rhizosphere and rhizosphere soils contaminated by heavy metals, which may be a signal of resistance to adversity (Zhang M. et al., 2022). In xenobiotics biodegradation and metabolism, the relative abundances of 11 functional genes were increased, and most of these heterologous metabolites were polluted by macromolecular organic compounds (such as organophosphorus, organochlorine, and aromatic hydrocarbons). Studies have shown that components including amino acids, microorganisms, and isoenzymes are combined with fertilizers and biological agents to produce products that degrade agricultural residues. This can achieve multi-purpose utility, such as the degradation of agricultural residues, yield increase, and improved stress resistance (Fei et al., 2021). In glycan biosynthesis and metabolism, peptidoglycan is the basic component of the bacterial cell wall and strengthens the cell wall, and lipopolysaccharide is the basic component of gram-negative bacterial cell walls. The relative abundances of the functional genes of biosynthesis of both decreased. It was speculated that with the application of plant-derived organic fertilizer, the physical properties of soil were improved, and the soil was not compact and had good air permeability. Bacteria no longer needed a hardened cell wall to survive.

In the function prediction of soil fungi, it was found that the relative abundance of saprophytes accounted for the majority in all treatments. Such organisms feed on dead organic matter and can decompose dead and weakened organic matter into simple substances for easy absorption and recycling by plants, which is beneficial to soil nutrient activation and crop growth and extremely important to soil ecosystems (Mahmud et al., 2017). In addition, it was found that the soil fungal communities exhibited the special functions of plant endophytes, plant saprophytes, and wood saprophytes due to the application of different botanical oil meals. Endophytic fungi live in plants, and this special environment makes it easier for these fungi to play a role in biocontrol compared to other saprophytes and epiphytes. Zhang L. et al. (2022) isolated 144 endophytes from different tissues of medicinal plants, among which strain 8ZJF-21 showed broad-spectrum antifungal activity and promoted plant growth by producing a variety of extracellular enzymes and metabolites. Wood saprophytes are regarded as forest cleaners. These fungi can decompose dead branches and fallen leaves and return them to nature, thereby participating in the material cycle and promoting the natural metabolism of forest trees to maintain ecological balance (Schwarze et al., 2022).

## 5. Conclusion

In this study, the mixed application of different botanical oil meals and cow manure increased the yield and quality of tobacco to different degrees compared with the single application of cow manure. Peanut bran had the best comprehensive effect, significantly improving the soil available-P, available-K, and NO_3_^–^-N. Furthermore, the addition of different botanical oil meals changed the composition of the soil microbial community. Bacteria such as *subgroup_7* and *Spingomonas* and fungi such as *Chaetomium* and *Penicillium* were enriched in the treatments of botanical oil meal mixed with cow manure, which are beneficial functional bacteria, indispensable in improving the ecological function of soil and protecting the safety of soil microecology. In addition, the prediction of soil bacterial community functions showed that sesame meal contributed greatly to the degradation and metabolism of heterologous organisms and could effectively reduce macromolecular organic compound pollution. The functional groups of endophytic fungi and wood saprophytes increased after the application of cow manure, which may enhance the broad-spectrum antibacterial activity of crops and maintain the soil’s ecological balance. As such, compared with adding cow manure alone, the mixed application of different botanical oil meals and cow manure solved the disadvantages of applying only one kind of organic manure, improved the physicochemical properties and enzyme activities of soil, increased the yield and quality of flue-cured tobacco, and promoted the soil microbial diversity and ecosystem stability.

## Data availability statement

The original contributions presented in this study are included in the article/Supplementary material, further inquiries can be directed to the corresponding authors.

## Author contributions

JW and BH: conceptualization and funding acquisition. YC and YQ: methodology. XL: software. DZ and CZ: validation. ZS, DL, and LJ: investigation. BH and YC: writing—original draft preparation. BH: writing—review and editing. LJ and JW: supervision. All authors have read and agreed to the published version of the manuscript.
