# Supplementary material for: Effects of different botanical oil meal mixed with cow manure organic fertilizers on soil microbial community and function and tobacco yield and quality
Source: Front Microbiol. 2023 May 25;14:1191059. doi: 10.3389/fmicb.2023.1191059 (PMC10248155; doi:10.3389/fmicb.2023.1191059)
Supplement: Supplementary file 1 [file Table_1.DOCX]

**Table S1: Main nutrient content of organic fertilizer**

| Types | Organic matter (%) | N (%) | P_2_O_5_ (%) | K_2_O (%) |
| --- | --- | --- | --- | --- |
| cow manure  rape meal  soybean meal | 45  42.5  88.0 | 0.45  4.8  7.0 | 0.5  2.1  1.1 | 0.3  1.9  2.1 |
| peanut bran | 47.9 | 6.3 | 1.2 | 1.3 |
| sesame meal | 22.4 | 5.8 | 3.0 | 1.3 |

**Table S2: Effects of different organic fertilizers on agronomic characters of tobacco**

| Treatments | Plant height  （cm） | Number of  blades | Maximum leaf  length（cm） | Maximum leaf  width（cm） | |
| --- | --- | --- | --- | --- | --- |
| NF | 123.40±3.39 a | 18.80±0.63 a | 69.20±1.84 a | | 26.87±1.10 b |
| NFC  NFD | 124.93±2.90 a  122.07±2.10 a | 18.93±0.46 a  18.67±0.57 a | 70.00±2.60 a  68.53±2.58 a | | 27.80±1.44 ab  26.97±1.13 b |
| NFHS | 122.20±3.28 a | 18.40±0.44 a | 72.40±3.02 a | | 29.53±1.27 a |
| NFZM | 121.40±2.80 a | 18.33±0.55 a | 69.33±1.73 a | | 27.33±0.99 b |

**Table S3: Effects of different organic fertilizers on yield and quality of flue-cured tobacco**

| Treatments | yield  （kg/hm^2^） | production（yuan/hm^2^） | Average price（yuan/ kg） | Quality tobacco ratio（%） |
| --- | --- | --- | --- | --- |
| NF | 1885.95 | 50431.8 | 26.74 | 59.16 |
| NFC | 2155.5 | 61562.1 | 28.56 | 71.01 |
| NFD | 2111.1 | 58947 | 27.92 | 83.54 |
| NFHS | 2419.5 | 65177.55 | 26.94 | 65.22 |
| NFZM | 2288.4 | 63208.2 | 27.62 | 79.59 |
